# Phylogenetic Analysis of West Nile Virus, Nuevo Leon State, Mexico

**DOI:** 10.3201/eid1007.030959

**Published:** 2004-07

**Authors:** Bradley J. Blitvich, Ildefonso Fernández-Salas, Juan F. Contreras-Cordero, María A. Loroño-Pino, Nicole L. Marlenee, Francisco J. Díaz, José I. González-Rojas, Nelson Obregón-Martínez, Jorge A. Chiu-García, William C. Black, Barry J. Beaty

**Affiliations:** * Colorado State University, Fort Collins, Colorado. USA;; † Universidad Autonoma de Nuevo Leon, Apartado, San Nicolas de los Garza, Nuevo Leon, Mexico;; ‡ Secretaria de Salud de Nuevo Leon, Nuevo Leon, Mexico

**Keywords:** West Nile virus, flavivirus, Mexico, phylogenetic analysis, genetic variation, horse, surveillance, dispatch

## Abstract

West Nile virus RNA was detected in brain tissue from a horse that died in June 2003 in Nuevo Leon State, Mexico. Nucleotide sequencing and phylogenetic analysis of the premembrane and envelope genes showed that the virus was most closely related to West Nile virus isolates collected in Texas in 2002.

West Nile virus (WNV), a mosquitoborne virus in the genus *Flavivirus* (family *Flaviviridae*), was first recognized in the Western Hemisphere during an outbreak in New York in 1999 (1). WNV rapidly disseminated across North America, and its geographic range now encompasses 47 of the 48 contiguous United States (2), 7 Canadian provinces (3), and several Mexican states (4–7).

A phylogenetic analysis of the prototype New York strain (WN-NY99-flamingo382-99), isolated from a dead flamingo from the Bronx Zoo in 1999, showed that this strain was most similar to an Israeli isolate from 1998 (8). WNV isolates collected in the northeastern United States in 2000 were similar to isolates collected in 1999 (9–12). However, studies performed with WNV isolates collected after 2000 suggest that genetically distinct populations have emerged in the United States (13,14). For example, up to 12 nucleotide substitutions (0.60% divergence) were present in the premembrane and envelope protein (prM-E) genes of isolates collected from inland and southeast coastal areas of Texas in 2002 (13).

More recently, Estrada-Franco et al. (5) reported the first isolation of WNV from Mexico. The isolate (TM171-03) was from a corvid that died on May 5, 2003, in Tabasco State, southern Mexico. We identified WNV RNA in the brain of a dead horse from Nuevo Leon State, northern Mexico. Nucleotide sequencing and phylogenetic analysis of the prM-E genes showed that this WNV from Mexico was most similar to isolates collected from noncoastal areas of Texas in 2002.

## The Study

Cerebellar tissue was taken from a dead 12-year-old stallion from a privately owned ranch in the municipality of Juarez in Nuevo Leon State, Mexico, approximately 240 km south of the Texas border. The horse was first observed with neurologic symptoms on June 20, 2003, and it was euthanized 7 days later. The horse had never been outside the state of Nuevo Leon and had not been vaccinated against WNV. The tissue sample was immediately placed on dry ice and transported to the biosafety-level-3 facilities at Colorado State University for processing. Although we were unable to isolate virus from the sample by passing brain homogenate in Vero cells, we successfully amplified viral RNA.

Total RNA was extracted from approximately 100 µg of cerebellar tissue with Trizol reagent (Invitrogen, Carlsbad, CA), according to the manufacturer's instructions. The prM-E genes were amplified as two fragments by reverse transcription-polymerase chain reaction (RT-PCR) by using primers designed from the nucleotide sequence of the prototype WN-NY99 strain (GenBank accession no. AF196835). PCR amplifications were performed by using Ex Taq DNA polymerase (Takara Biomedicals, Shiga, Japan), which has 3´ → 5´ exonuclease activity. Amplification products were separated by agarose gel electrophoresis, visualized with crystal violet, and extracted by using the rapid gel extraction system (Invitrogen, Carlsbad, CA). The resulting DNA fragments were reamplified by PCR because of the low RNA copy number in the original material and purified by using the QIAquick PCR purification kit (Qiagen, Valencia, CA). Purified DNAs were sequenced on both strands with an ABI 377 DNA sequencer (Davis Sequencing, Davis, CA) and eight pairs of WNV-specific primers.

The nucleotide sequence of the prM-E genes of the WNV from Nuevo Leon State, Mexico (designated MexNL-03) was submitted to GenBank (GenBank accession no. AY426741). This region comprises 2004 nucleotides and corresponds to nucleotides 466 to 2469 of the genomic RNA of the WN-NY99 strain (8). Alignment of the MexNL-03 sequence with other known sequences in the GenBank database showed that it was most closely related to the homologous regions of three WNV isolates collected in Harris County, Texas, in June 2002 (strains 119, 123, and V1151; GenBank accession numbers AY185908, AY185909, AY185911 respectively). The MexNL-03 sequence differed from the Harris County isolates in three nucleotide positions (0.15% divergence; Table). In all cases, one change was in the prM gene at position 549, and two changes were in the E gene at positions 1179 and 1356. All substitutions were in the third codon position, and none resulted in an amino acid change.

**Table:** Nucleotide and deduced amino acid differences in the premembrane and envelope genes of the West Nile virus from Nuevo Leon State compared with various other West Nile viruses

Strain	Geographic origin	Nucleotide no.^a^														
		483	549	660	858	887	1137	1179	1356	1432	1442	1626	2328	2388	2466	Ref.
WN-NY99^b^	New York, NY	C	U	C	C	U (Ile)^c^	C	A	C	U (Ser)	U (Val)	C	C	C	C	8
MexNL-03^d^	Nuevo Leon State, northern Mexico		C	U				G	U		C (Ala)				U	
TM171-03^e^	Tabasco State,southern Mexico	U			U	C (Thr)	U			C (Pro)		U	U	U	U	5
119^f^	Harris Co., inland Texas			U							C (Ala)				U	13

^a^Nucleotide numbers correspond to those of the prototype New York strain (WN-NY99). ^b^Isolated from a Chilean flamingo (*Phoenicopterus chilensis*) (collection date: 06/01/99). GenBank accession no.: AF196835. ^c^Amino acid changes are shown in parentheses. Gaps indicate no change in the nucleotide sequence. ^d^Isolated from a horse (collection date: 06/27/03). GenBank accession no.: AY426741. ^e^Isolated from Common Raven (collection date: 05/05/03). GenBank accession no.: AY371271. ^f^Isolated from a bluejay (*Cyanocitta cristata*) and passaged once in Vero cells (collection date: 06/14/02). GenBank accession no. AY185908.

The nucleotide sequence of MexNL-03 differed from that of the WN-NY99 strain (GenBank accession no. AF196835) in six positions (0.30% divergence; Table). Two mutations were in the prM gene (positions 549 and 660), and four mutations were in the E gene (positions 1179, 1356, 1442, and 2466). The U to C substitution at 1442 resulted in an amino acid change (Val → Ala); all other substitutions were silent. The U to C substitution at 549 and A to G substitution at 1179 have not been reported in any WNV isolates from the United States. However, an isolate collected in Illinois in 2002 (GenBank accession no. AY428521) has a U to A substitution at position 549. Similarly, an isolate from Randall County, Texas (GenBank accession no. AY428519), has an A to C substitution at position 1179. Several more divergent strains of WNV, such as a Kunjin virus isolated in Australia in 1960 (GenBank accession no. D00246), have a G at position 1179. The prM-E genes of MexNL-03 differed from TM171-03 (GenBank accession no. AY371271) in 13 nucleotide positions (0.65% divergence; Table). Five mutations were in the prM gene, and eight mutations were in the E gene. Three mutations resulted in amino acid changes.

**Figure 1:**
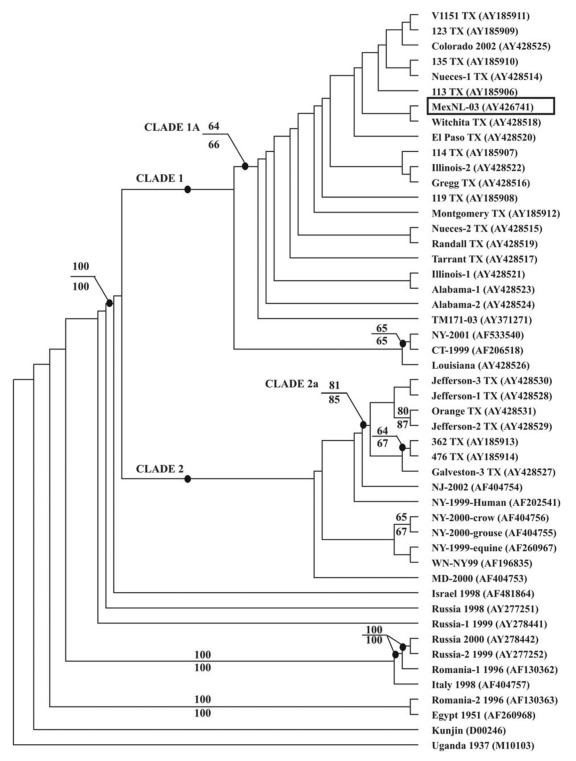
Phylogenetic analysis of West Nile virus (WNV) from Nuevo Leon State, Mexico. Phylogenies were estimated by using the program MRBAYES, version 2.0 (15). Sampling of trees from the posterior probability distribution used the Metropolis-coupled Markov chain Monte Carlo algorithm to allow running of multiple Markov chains. A run with four chains was performed for 90,000 generations, under a general time-reversible model (all six types of substitutions occur at different rates) with parameter value estimation for base frequencies, substitution matrix values, and rate heterogeneity. Rate heterogeneity was estimated by using a γ distribution for the variable sites and assuming a certain portion of sites to be invariable. The burn-in time was 70,000 generations. The phylogenetic analysis is based on the 2004-nt fragment encoding the complete prM-E genes of 49 WNVs. The tree is rooted by using the prototype WNV strain from Uganda in 1937 (GenBank accession no. M10103) as an outgroup. Values above some branches represent the percentage support by parsimony bootstrap analysis. Values below some branches represent the percentage support by distance bootstrap analysis. The bootstrap confidence estimates are based on 1,000 replicates. The WNV from Nuevo Leon State is encapsulated.

A phylogenetic tree was constructed by Bayesian analysis using the complete prM-E gene sequences of 49 WNV strains, including this WNV from Nuevo Leon State, Mexico (Figure 1). Phylogenetic trees were also generated using neighbor-joining (NJ), maximum parsimony (MP), and maximum likelihood (ML) analyses (data not shown). In the Bayesian tree, the WNV isolates from North America formed a monophyletic group consisting of two sister clades (denoted as clade 1 and 2). MexNL-03 shared a close phylogenetic relationship with isolates from inland Texas, consistent with our nucleotide sequence alignments. These viruses, along with isolates from Colorado, Illinois, Alabama, and Tabasco State, belonged to a nested clade (denoted as 1A) within clade 1. The statistical support for clade 1A by parsimony and distance bootstrap analyses were 64% and 66%, respectively. The other WNV isolates that clustered in clade 1 were from the eastern United States. Clade 2 contained WNV isolates from the southeast coastal area of Texas and the northeastern United States. The WNV isolates from coastal Texas formed a nested clade (denoted as 2A), confirming previous results (13,14).

**Figure 2:**
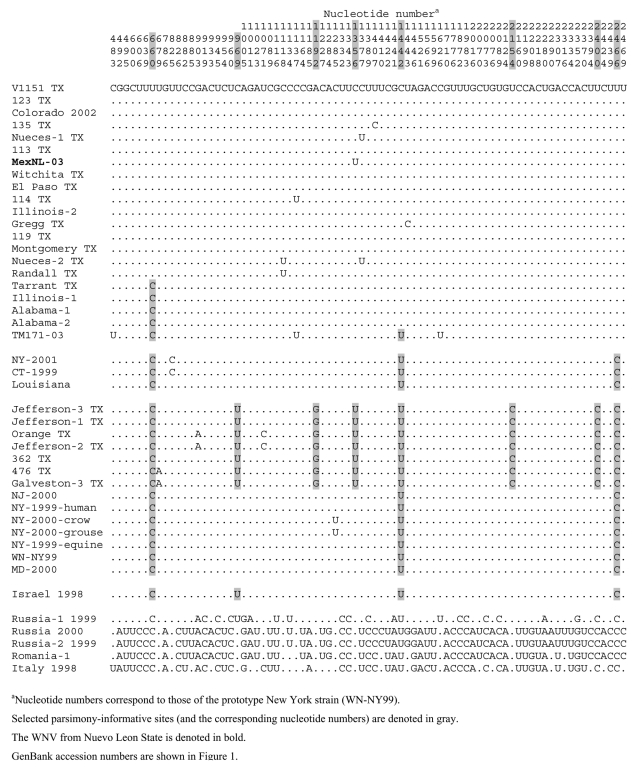
Parsimony-informative sites in the premembrane and envelope genes of selected West Nile viruses used in the phylogenetic analyses.

The parsimony-informative sites, corresponding to nucleotide positions 660, 1442, and 2466, were important in defining the topologic features of the tree (Figure 2). All 21 WNV strains in clade 1A contained a unique C to U substitution at 2466. All, except for the five most basal isolates, contained a unique C to T substitution at 660 and all, except for TM171-03, contained a unique T to C substitution at 1442. TM171-03 was basal to the other WNV strains in clade 1A, and this topologic arrangement was due to the single nucleotide difference at 1442. However, we consider it unlikely that TM171-03 was the predecessor of the other viruses in clade 1A. In addition, the parsimony and distance bootstrap analyses did not significantly support this topologic arrangement (bootstrap values = 50% and 51%, respectively). Moreover, WNV isolates from the northeastern United States occupied the basal position of clade 1. Similarly, the basal position of clade 2 was occupied by WNV isolates from the northeastern United States. Thus, our findings suggest that the WNV isolates circulating in the United States and Mexico diverged from a common ancestor from the northeastern United States.

The trees generated by NJ, MP, and ML analyses showed the same overall topologic features to the Bayesian tree, except that all of the coastal Texas isolates were basal to the Israeli isolate (data not shown). The bootstrap support for this topologic arrangement ranged from 58% to 73%. Similar findings were also reported by Estrada-Franco et al. (5). Furthermore, the WNV isolates from Maryland and New Jersey, as well as most of the isolates from New York, occupy the basal positions of clade 1 of the NJ/MP/ML trees. As a result, we have not shown the bootstrap values for clades 1 and 2 of the Bayesian tree because their composition does not match exactly to the corresponding clades of the NJ/MP/ML trees. However, the Bayesian analysis provides a more robust and efficient phylogenetic tool compared to more conventional phylogenetic techniques (15). Additional sequencing and phylogenetic analyses will be necessary to clarify these issues.

## Conclusions

The data presented here indicate that WNV was introduced into Nuevo Leon State, Mexico, from inland Texas. A likely mode of introduction was by infected birds traveling for short distances (16). Earlier studies have provided serologic evidence of WNV infection in horses or birds in the nearby Mexican states of Coahuila, Tamaulipas, and Chihuahua (4,5,17). Taken together, our sequence data and the findings from the serosurveys indicate that WNV activity is now widespread in northern Mexico, as well as in other regions in Mexico (5,6,18). The geographic distribution of WNV in the Americas will likely continue to expand; thus, enhanced WNV surveillance in Mexico is warranted.
